# Experimental Study on the Health Benefits of Garden Landscape

**DOI:** 10.3390/ijerph14070829

**Published:** 2017-07-24

**Authors:** Juyoung Lee

**Affiliations:** Department of Landscape Architecture, Hankyong National University, 327 Chungang-ro, Anseong-si, Gyeonggi-do 17579, Korea; lohawi@gmail.com; Tel.: +82-31-670-5213; Fax: +82-31-670-5219

**Keywords:** traditional garden, brain activity, autonomic nervous activity, winter landscape, individual difference

## Abstract

To mitigate the negative effects of modern cities on health, scientists are focusing on the diverse benefits of natural environments; a conceptual approach to use gardens for promoting human health is being attempted. In this study, the effects of the visual landscape of a traditional garden on psychological and physiological activities were investigated. Eighteen male and female adults participated in this indoor experiment (mean age, 26.7 years). Twelve different landscape images for city and garden were presented continuously for 90 s. In the time series changes of oxygenated hemoglobin (O_2_Hb), different patterns of changes were observed between the city and garden. The mean O_2_Hb values increased for the city landscapes, whereas they decreased for the garden landscapes both in the left and right prefrontal cortices. Significant differences in the negative psychological states of tension, fatigue, confusion, and anxiety were observed between the city and garden landscapes. Important differences in the physiological and psychological responses to the two different landscapes were also detected between male and female participants, providing valuable clues to individual differences in the health benefits of natural landscapes. To validate the use of gardens as a resource for promoting health in urban dwellers, further scientific evidence, active communication, and collaboration among experts in the relevant field are necessary.

## 1. Introduction

Over the past several decades, the level of urbanization in Asian countries has increased from 27% in 1950 to 57% in 2014 [1]. Currently, Korea is considered one of the most urbanized countries (82%). However, urbanization does not necessarily improve the quality of life [2]. Despite the many advantages of developed infrastructures, artificialized urban environments, in general, might be closely related to the negative health outcomes in modern people, which is supported by an increasing body of research [3,4,5,6].

To mitigate the negative effects of modern city environments, scientists are currently focusing on the benefits of natural environments [7,8,9]. In recent years, quantified scientific evidence has been reported on the health benefits of natural environments with the development of biological markers [10,11,12,13,14,15]. The epidemiological approach supports the positive relationship between nature and health-related parameters [16,17]. On the basis of these findings, evidence-based policy movement is also undertaken. The Korean government passed legislation to use forests for promoting health, which included national funding support from the government, a specialist production system, and facility construction on forest therapy [18].

The visible landscape is believed to affect human beings in many ways, including aesthetic appreciation, health, and well-being [19]. Since the landmark study of Ulrich [20] that demonstrated that window landscape views affect patient recovery after surgery, increasing attention has been paid to the health-related effects of visible landscape in the fields of landscape architecture and public health [19,21,22,23].

A new conceptual approach is being developed to use gardens for promoting human health [24,25,26,27,28]. Gardens are private or public green spaces ornamented with vegetation and beautiful landscapes that are used for recreation, restoration, and healing. In recent years, there has been a growing awareness of the need for gardens for stress reduction and healing [29,30,31]. Landscape features in gardens vary with scales and styles. Among these, traditional gardens often represent local identities [32] by revealing common landscape features of local areas and providing activity spaces for urban dwellers [33]. However, despite increasing social needs, scientific evidence on the health benefits of garden landscapes is still limited to support this notion. Therefore, this study aimed to investigate the effects of visual landscapes of traditional gardens on psychological and physiological activities by quantifying health-related benefits.

## 2. Methodology

### 2.1. Participants and Landscape Images

The voluntary participants were 18 young adults consisting of 9 male and 9 female with a mean age (±standard error (SE)) of 26.7 ± 0.7 years (Table 1). All participants were physically and psychologically healthy. Those with past and/or current mental disorders, cardiovascular or allergic diseases, and smoking or drinking habits were screened in the process of recruiting. Prior to the experiment, written informed consent was obtained from all the participants after the study aims and protocol were provided and explained. This study was approved by the Ethics Committee (08-10) of the Graduate School of Horticulture, Chiba University.

**Figure 1:**
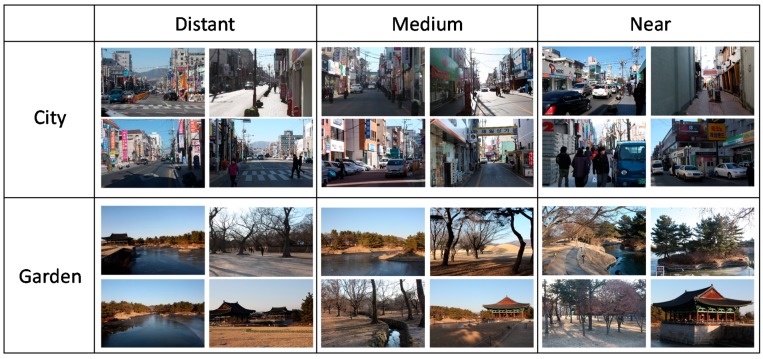
Landscape images of city and garden used in the experiment.

City and garden landscape images were prepared by taking pictures in Gyeongju city in Korea, which has some of the most famous historic places with a vast number of archaeological sites and traditional properties. Some parts of the city have even been designated as world heritage sites by the UNESCO. City landscape images were taken at the commercial area of the city center, which exhibits typical characteristics of the local cities in Korea. Garden landscape images were taken at the Anapji, one of the typical traditional gardens in Korea. Twelve different views in each area were used in the experiment and were taken at three different distances, i.e., distant, medium, and near. Pictures were taken in the winter season to evaluate the health-related benefits of winter landscapes (Figure 1). Each city or garden session lasted for 90 s with 12 images shown in total. Each person viewed both the city and garden images and the order of the sessions was counterbalanced for each participant. Before the experimental sessions, dummy images were also provided. Images were presented using a high definition display placed 3 m from the participant. The room temperature was set at 25 °C and relative humidity at 60%.

### 2.2. Data Collection

As an index of the physiological response, blood flow in the brain was monitored using near-infrared spectroscopy (NIRS) to investigate the changes in brain activity when observing two different landscapes. Hemoglobin concentrations in the left and right prefrontal cortices were continually measured using NIRO-300 (Hamamatsu Photonics, Shizuoka, Japan) with 1-s segments during the experiment. Increased brain activity was accompanied by increased blood flow, because brain activity requires oxygen consumption. It has been known that the blood flow changes are consistent with changes of oxy-hemoglobin (O_2_Hb) [34], and that decreases in O_2_Hb concentrations are related to physiological relaxation [35]. Prior to the viewing session, the O_2_Hb concentration in the left and right prefrontal cortices was confirmed to have become constant for 15 s. To investigate the reactivity of the autonomic nervous system, systolic and diastolic blood pressures and pulse rate were measured with an oscillometric method (HEM-1010, Omron, Kyoto, Japan) following the presentation of each landscape image.

The Profile of Mood States (POMS) [36] was used to assess the following six mood dimensions on a five-point scale: “tension-anxiety (T-A)”, “depression (D)”, “anger-hostility (A-H)”, “confusion (C)”, “vigor (V)”, and “fatigue (F)”. The Total Mood Disturbance (TMD) score was also calculated for the POMS data. A semantic differential (SD) method [37] was used to evaluate the emotional response to the city and garden landscapes. The SD rating test was conducted on seven scales for eight different pairs of adjectives, including natural-artificial, open-closed, attractive for walking-unattractive for walking, comfortable–uncomfortable, familiar-unfamiliar, clean-dirty, beautiful-ugly, and warm-cold. To compare the changes in the level of state anxiety for each landscape, the State-Trait Anxiety Inventory (STAI) [38] test was also conducted. Three types of psychological tests were administered to the participants after they watched the landscape images.

### 2.3. Data Analysis

For each 1-s segment of NIRS data, time series changes were investigated and differences at each time segment between city and garden landscapes were analyzed. Hemoglobin concentrations were also calculated on three 30-s periods—early, mid, and late—to compare male and female hemodynamic responses to the two landscapes. Systolic and diastolic blood pressures were also measured to investigate the autonomic nervous reactivity to the landscape images. The physiological results were analyzed using the paired *t*-test and the psychological data were compared by the Wilcoxon signed-rank test. Statistical analysis was conducted using SPSS 21.0 (IBM Corporation, Armonk, NY, USA). All data were presented as means ± standard error (SE) and the statistical differences were considered to be significant at *p* < 0.05.

## 3. Results

### 3.1. Cerebral Blood Flow

**Figure 2:**
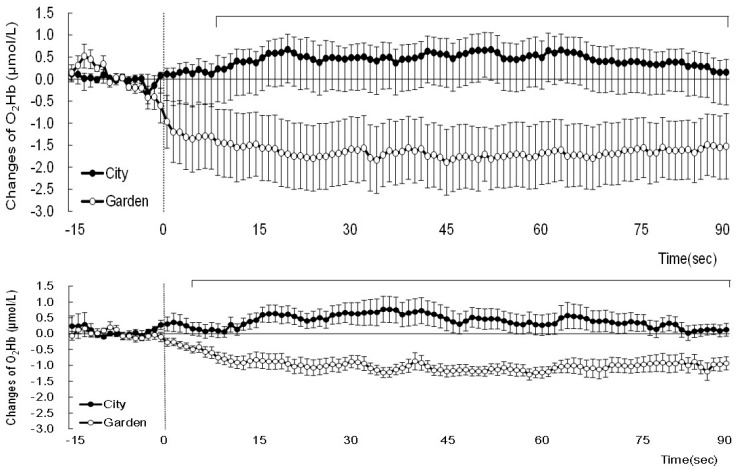
Changes in oxy-hemoglobin (O_2_Hb) in the left (**top**) and right (**bottom**) prefrontal cortex areas when observing city and garden landscapes. Mean ± standard error; *N* = 18; bar means significant difference.

**Figure 3:**
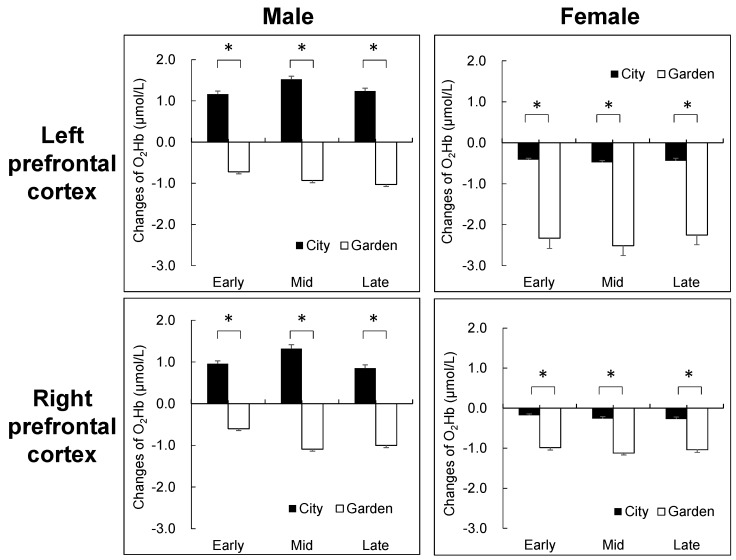
Comparison of oxy-hemoglobin (O_2_Hb) concentrations between male and female participants when observing city and garden landscapes in early, mid, and late sessions. Mean ± standard error; Male *N* = 9; Female *N* = 9; * *p* < 0.05.

The NIRS data revealed different hemodymanic reactivity to the city and garden landscapes. At first, there were no significant differences between the two stimuli in the left and right hemispheres before viewing the images of each landscape. Mean values of O_2_Hb for 90 s of city stimuli were 0.43 nmol/L (±0.03) and 0.40 nmol/L (±0.03) in the left and right hemispheres, respectively. In contrast, the mean values of O_2_Hb for 90 s of garden stimuli were decreased to −1.63 nmol/L (±0.07) and −0.97 nmol/L (±0.02) in the left and right hemispheres, respectively. In the time series changes of O_2_Hb, different patterns of changes were found between the city and garden (Figure 2). The mean O_2_Hb values increased for the city landscape, whereas they decreased for the garden both in the left and right prefrontal cortices. Significant differences were detected during 8 to 90 s in the left hemisphere and during 5 to 90 s in the right hemisphere for the garden images, compared with the city landscape images. The O_2_Hb values of the garden landscape were significantly lower from 8 to 90 s in the left hemisphere and 5 to 90 s in the right hemisphere, compared with the city landscape images. In the analysis of the three 30-s values, significant differences were detected between the city and garden landscapes for the early, mid, and late time periods. Interesting differences were also found in the mean values of the O_2_Hb concentrations between male and female participants (Figure 3). Although the female participants exhibited a decrease for both landscapes, the male participants displayed an apparent increase for the city landscape and a decrease for the garden, indicating more distinguished differences for the city than the garden.

### 3.2. Autonomic Nervous Activity

**Figure 4:**
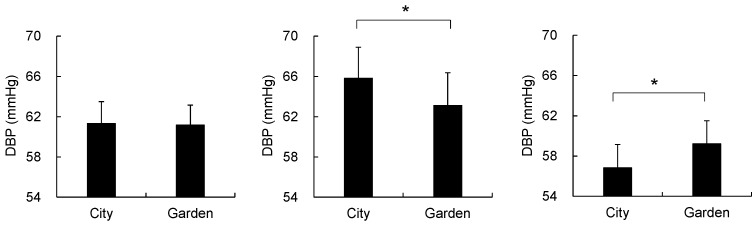
Comparison of the diastolic blood pressure (DBP) between city and garden landscapes in all (**left**), male (**middle**), and female (**right**) participants. Mean ± standard error; Total *N* = 18; Male *N* = 9, Female *N* = 9; * *p* < 0.05.

No significant differences between the city and garden landscapes were found in the analysis of systolic (101.1 ± 2.9 mmHg for city landscape; 101.7 ± 2.6 mmHg for garden landscape) and diastolic blood pressures (61.3 ± 2.2 mmHg for city landscape; 61.2 ± 2.0 mmHg for garden landscape); the pulse rate (67.4 ± 1.6 bpm for city landscape; 66.7 ± 1.7 bpm for garden landscape) was also similar for both landscapes. However, when comparing between the male and female participants, significant differences were observed in the diastolic blood pressure (Figure 4). Male participants had a significantly higher value for the city (65.8 ± 3.1 mmHg) than for the garden (63.1 ± 3.3 mmHg; *p* < 0.05) landscape, while the female participants exhibited a significantly lower value for the city (56.8 ± 2.3 mmHg) than for the garden (59.2 ± 2.3 mmHg; *p* < 0.05) landscape. For the systolic blood pressure and pulse rate, both male and female participants displayed similar responses to the city and garden landscapes with no significant differences.

### 3.3. Psychological Response

**Figure 5:**
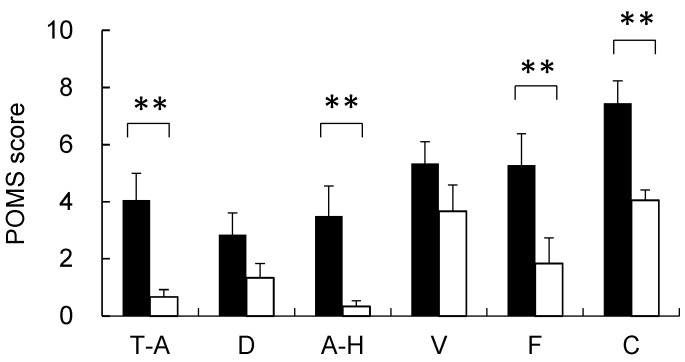
Comparison of the profile of mood states (POMS) scores between city and garden landscapes. T-A, tension-anxiety; D, depression; A-H, anger-hostility; F, fatigue; C, confusion; V, vigor; Mean ± standard error; *N* = 18; ** *p* < 0.01.

**Figure 6:**
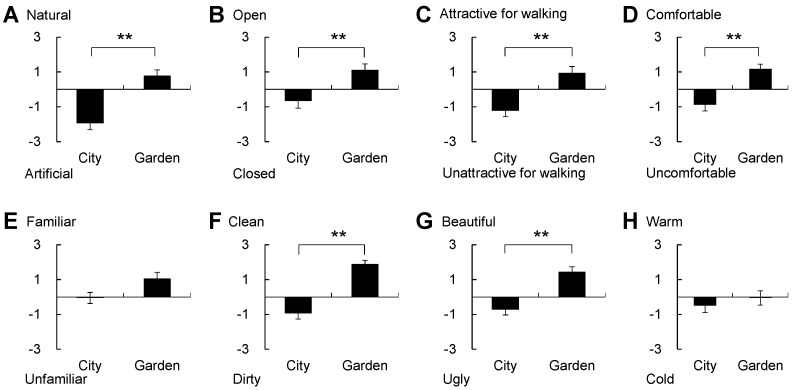
Comparison of the semantic differential (SD) scores for eight different feelings between city and garden landscapes. Subfigures indicate the feelings of natural-artificial (**A**), open-closed (**B**), attractive for walking-unattractive for walking (**C**), comfortable–uncomfortable (**D**), familiar-unfamiliar (**E**), clean-dirty (**F**), beautiful-ugly (**G**) and warm-cold (**H**). Mean ± standard error; *N* = 18; ** *p* < 0.01.

**Figure 7:**
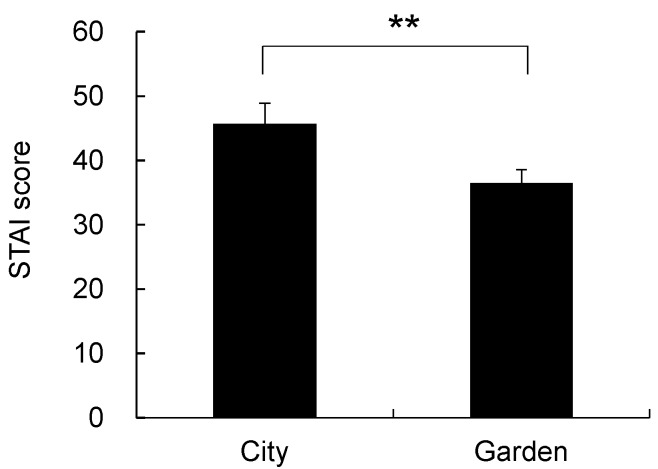
Comparison of the state-trait anxiety inventory (STAI) scores between city and garden landscapes. Mean ± standard error; *N* = 18; ** *p* < 0.01.

In the analysis of the psychological responses to the images of the two landscapes, significant differences were detected. In the POMS analysis (Figure 5), significant differences were found between the city and garden landscapes for negative mood states of T-A (city, 37.2 ± 1.4; garden, 32.1 ± 0.4; *p* < 0.01), A-H (city, 40.7 ± 1.1; garden, 37.3 ± 0.2; *p* < 0.01), F (city, 42.2 ± 1.7; garden, 36.9 ± 1.4; *p* < 0.01), C (city, 45.8 ± 1.5; garden, 39.1 ± 0.7; *p* < 0.01), and TMD (city, 172.1 ± 5.8; garden, 152.9 ± 3.2; *p* < 0.01). Based on the SD data for the city and garden landscapes (Figure 6), the garden was evaluated as significantly more natural (city, −1.94 ± 0.37; garden, 0.78 ± 0.34; *p* < 0.01), more open (city, −0.67 ± 0.41; garden, 1.11 ± 0.35; *p* < 0.01), more attractive for walking (city, −1.22 ± 0.35; garden, 0.94 ± 0.37; *p* < 0.01), more comfortable (city, −0.89 ± 0.35; garden, 1.17 ± 0.28; *p* < 0.01), cleaner (city, −0.94 ± 0.33; garden, 1.89 ± 0.21; *p* < 0.01), and more beautiful (city, −0.72 ± 0.31; garden, 1.44 ± 0.29; *p* < 0.01), compared with the city landscape. A significant difference was also observed in the state anxiety level (Figure 7) between the city (45.7 ± 3.2) and garden (36.5 ± 2.1; *p* < 0.01) landscapes.

When comparing the POMS scores between male and female participants, a significant difference was found in the T-A subscale. The male group, but not the female, exhibited a significant difference in the T-A scores between the city and garden. The scores of the negative subscales in the female group (T-A, 36.1 ± 2.0; D, 40.9 ± 0.8; A-H, 40.2 ± 1.6; F, 40.0 ± 2.2; C, 44.1 ± 1.6; TMD, 164.4 ± 7.3) were lower than in the male group (T-A, 38.3 ± 2.0; D, 42.4 ± 1.2; A-H, 41.2 ± 1.7; F, 44.4 ± 2.5; C, 47.4 ± 2.5; TMD, 179.8 ± 8.7) for the city landscape.

## 4. Discussion

In the present study, the physiological and psychological responses to the city and garden landscapes were investigated in healthy male and female adults. There were significant differences in the cerebral activity between the two landscapes. For the garden landscape, the cerebral activity was slowly decreased as evidenced by the declining O_2_Hb concentrations in the prefrontal areas, compared with the city landscape. This result can be related to decreased physiological stress. In the analysis of psychological responses, negative mood states—including tension, anger, fatigue, confusion, and anxiety—were significantly reduced when viewing the garden compared with the city scenes. Although similar results have been shown in previous studies on natural environments, including forest [11,12,39], farmland [40], urban park [41], and neighborhood green space [42], only a few studies have reported the health-related value of traditional gardens. The traditional garden has unique landscape features different from other green spaces such as forests or parks, because it includes historical and cultural elements in its view. Therefore, the evidence derived from this study might support the new function of traditional gardens for the health and well-being of urban dwellers.

Although an emerging body of research is focused on the diverse benefits of natural landscapes, very little has been considered on the effects of winter landscapes. Song et al. [43] investigated the effects of walking in urban parks in winter and found positive outcomes on the cardiovascular activity and psychological states. These outcomes reflected not only the effects of environmental stimuli but also the results of physical activity, which has limitations in considering the benefits of visual winter landscapes. Therefore, the present results may support the fact that these health benefits arise from the natural landscape itself even in the absence of lush green vegetation.

Important differences in the physiological and psychological responses to the two different landscapes were also detected between male and female groups. In 30-s segment NIRS data, the female group exhibited different patterns of O_2_Hb changes than the male group. When viewing the city landscape, the mean O_2_Hb values increased in the male group and slightly decreased in the female group, displaying a more significant difference in the left prefrontal area than in the right area. Similar trends were also observed in the diastolic blood pressure results. After viewing the two landscape images, the female group exhibited significantly lower values for the city, while the male group displayed higher values for the city. The women might have had a less negative perception of the city landscape than the male participants, because self-reported psychological tests indicated that the female group had lower levels of negative mood states related to the city than males. On the basis of these sex differences in multiple parameters, it is suggested that sex might be a factor for characterizing the individual differences for landscape preferences, as previously suggested by Lyons [44].

Despite the importance of individual differences in association with health benefits of natural environments, there is still not enough research-based evidence. Individual differences can diverge in the participants’ attributes, such as age, sex, socioeconomic status, and environmental experience [44,45]. Indeed, the results of this study revealed different health outcomes of two different landscapes between male and female participants. The data from the female participants might be partly inconsistent with previous studies showing the negative effects of city environments in young adults. Recent studies dealt with this issue from the view point of Type A personality or law of initial value [46,47]. In addition to these previous studies, the present experimental study provided valuable clues to individual differences in the health benefits of natural landscapes, despite the limitation of the small sample size. Solid evidence on this issue is still lacking and further studies are needed to advance natural environment as a health promoting agent.

Because of the increasing interest in their health benefits, there are many initiatives advocating the use of natural environments for promoting health, such as the Green Gym project [48]. The Natural Environment Initiative [49] was launched by Harvard University to investigate how natural environments can support human health and well-being through the participation of many specialists including public health scientists, landscape architects, and environmental psychologists. In Japan, the forest therapy project has been carried out to utilize the forest environment as a health promotion agent throughout the country. National movement is also undertaken in Korea through the reform of public policy and legislation to deal with the health problems of modern people, including depression and lifestyle-related diseases. From this perspective, traditional gardens may need to be considered as one of the health promoting resources for urban dwellers as well as historic and cultural resources. To materialize this idea, scientifically rigorous evidence is still required through further studies and active communication and collaboration among experts in the fields of public health, cultural properties, landscape architecture, and policymaking.

## 5. Conclusions

In the present study, the physiological and psychological responses to the city and garden landscapes were investigated in healthy male and female adults. For the garden landscape, the cerebral activity was decreased as evidenced by the declining O_2_Hb concentrations in the prefrontal areas. Negative mood states—including tension, anger, fatigue, confusion, and anxiety—were significantly reduced when viewing the garden compared with the city scenes. Differences in the physiological and psychological responses to the two different landscapes were detected between male and female groups.

When viewing the city landscape, the mean O_2_Hb values increased in the male group and slightly decreased in the female group. In the analysis of the diastolic blood pressure for the city landscape, the female group exhibited significantly lower values, while the male group displayed higher values. In multiple parameters, the female group exhibited less negative or slightly positive responses to the city landscape than the male group, suggesting that sex might be a factor for characterizing the individual differences for landscape preferences. Despite an emerging body of research in this field, the effects of winter landscapes have been rarely considered. The present study revealed a part of health-related values of winter landscape, supporting that these health benefits stem from the natural element itself even in the absence of lush green vegetation. The evidence derived from this study might support the new function of traditional gardens for the health and well-being of urban dwellers.

## Figures and Tables

**Table 1:** Baseline values of the participants.

Parameters	Total ^a^		Male ^a^		Female ^a^	
	Mean	SE	Mean	SE	Mean	SE
Age	26.7	0.7	27.8	1.1	25.6	0.8
Height (cm)	167.4	1.3	171.2	1.0	163.7	1.5
Weight (kg)	57.7	1.7	62.8	1.4	52.6	1.9
BMI (kg/m^2^) ^b^	20.5	0.4	21.4	0.5	19.6	0.5
Pulse rate (bpm)	67.6	1.7	69.1	3.1	66.2	1.5
SBP (mmHg) ^b^	107.9	2.7	115.8	3.4	100.1	1.9
DBP (mmHg) ^b^	64.4	1.9	66.0	3.3	62.8	2.0

^a^ Total sample *N* = 18; Male sample *N* = 9; Female sample *N* = 9; ^b^ BMI, body mass index; SBP, systolic blood pressure; DBP, diastolic blood pressure.
